# Taxonomic revision of the *Asplenium wrightii* complex (Aspleniaceae) with reinstatement of *A. alatulum* and *A. subcrenatum*

**DOI:** 10.3897/phytokeys.172.62511

**Published:** 2021-02-12

**Authors:** Ke-Wang Xu, Lu-Lu Wang, Li-Bing Zhang

**Affiliations:** 1 Co-Innovation Center for Sustainable Forestry in Southern China, College of Biology and the Environment, Nanjing Forestry University, Nanjing; 510275, China Nanjing Forestry University Nanjing China; 2 Missouri Botanical Garden, P.O. Box 299, St. Louis, Missouri 63166-0299, USA Missouri Botanical Garden St. Louis China

**Keywords:** species delimitation, spore morphology, stomata

## Abstract

The *Asplenium wrightii* complex is morphologically variable and difficult in species delimitation. Owing to lack of comprehensive sampling in phylogenetic studies, the taxonomy of this complex remains unresolved. Based on extensive field observations, specimen examination and our recent molecular data, the present study aims to clarify the identities of three species of *Asplenium* in this complex from Asia. Our study revealed that *A. alatulum* and *A. subcrenatum*, previously treated as synonyms of *A. wrightii*, should be reinstated. A taxonomic revision of the three species, including their type information, detailed descriptions, voucher specimens, distribution, ecology, as well as taxonomic notes, is carried out.

## Introduction

*Asplenium* L. (Aspleniaceae) comprises more than 700 species of epilithic, epiphytic or terrestrial herbs, distributed throughout temperate and tropical regions of the world (Kramer and Viane 1990). A number of systematists worked on the taxonomy of the genus, based on morphological characteristics (Presl 1836; Hooker and Baker 1874; Christ 1897; Hayata 1927; Copeland 1947; Holttum 1947; Nayar 1970; Iwatsuki 1975; Mickel 1976; Pichi Sermolli 1977; Tryon and Tryon 1982; Bir et al. 1985; Wu 1989; Kramer and Viane 1990; Lin and Viane 2013). In the late 1980s, Wu (1989) published a comprehensive treatment of the genus in China in which four sections and five series were recognised. Asplenium ser. Wrightiana Ching & S.H.Wu is one of the five series in A. sect. Asplenium. Members of this series have 1-pinnate laminae, often falcate pinnae and serrate pinna margins. The wide range of morphological variation in A. ser. Wrightiana in China has been the basis of different taxonomic treatments. Wu (1999) included 14 species in A. ser. Wrightiana from China (Table 1), while Lin and Viane (2013) only recognised four species (*A. finlaysonianum* Wall. ex Hook, *A. loriceum* Christ, *A. matsumurae* Christ and *A. wrightii* Eaton ex Hooker) and treated others as synonyms of *A. wrightii* (Table 1). However, *A. finlaysonianum*, *A. loriceum* and *A. matsumurae* were found not to be closely related to the *A. wrightii* complex in a phylogenetic study (Xu et al. 2020). The *A. wrightii* complex was nested within *A. bullatum* clade, while species *A. finlaysonianum* was nested within *Tarachia* clade and species *A. loriceum* and *A. matsumurae* were nested within *Neottopteris* clade (Xu et al. 2020). Therefore, these three species, *A. finlaysonianum*, *A. loriceum* and *A. matsumurae* were excluded from the *A. wrightii* complex.

*Asplenium alatulum* Ching was first published, based on a single collection from Wuzhishan Mountain in south-central Hainan, China in 1922. In the protologue, Ching and Wang (1964) described *A. alatulum* as a small herb with short pinnae, pinna apices acuminate, pinna margins crenate-serrate and rachises with lateral wings. *Asplenium subcrenatum* Ching ex S. H. Wu (1989) was based on the type material from Malipo County, south-eastern Yunnan, China. He stated that *A. subcrenatum* is similar to *A. wrightioides* Christ, but the stipe and the rachis of the former species are densely covered with red fibrillar scales. These two species were recognised by Wu (1999), but not by Lin and Viane (2013), who treated them as synonyms of *A. wrightii* in light of variable morphological characters within this aggregate. Lin and Viane (2013) further artificially separated the *A. wrightii* complex into four groups, based on a set of morphological characters, such as the venation and the length of the pinnae and the length of sori.

During our taxonomic study on *Asplenium*, we found that *A. alatum*, *A. subcrenatum* and *A. wrightii* could be easily identified and are distinguishable from one another, based on morphological characteristics and geographical distribution. Our recent global phylogeny of *Asplenium* (Xu et al. 2020) supports both *A. alatulum* and *A. subcrenatum* as not conspecific with *A. wrightii*. The *A. wrightii* aggregates were not resolved as monophyletic because the clade also included A. × shikokianum, a hybrid between *A. wrightii* and *A. ritoense* (Xu et al. 2020) which is not a member of the *A. wrightii* aggregates sensu Wu (1989). In the present study, we evaluate the morphological characteristics of spores and scales of *A. alatulum*, *A. subcrenatum* and *A. wrightii*, in combination with our earlier molecular work, to establish the delimitation and validity of the two species and to produce a taxonomic treatment including descriptions and distributional notes.

**Table 1.** List of the 14 species included in Asplenium ser. Wrightiana by Wu (1999) in alphabetical order, references being given in the right-hand column. Species indicated by asterisk were treated as synonyms of *A. wrightii* by Lin and Viane (2013). Four species, recognised by Lin and Viane (2013), are indicated with boldface.

Species	Reference
*Asplenium alatulum* Ching*	Acta Phytotax. Sinica 9 (4): 359. 1964
*A. duplicatoserratum* Ching ex S. H. Wu*	Bull. Bot. Res. 9 (2): 19, f. 4. 1989.
***A. finlaysonianum* Wall. ex Hook.**	Ic. Pl. t. 937. 1854
*A. fujianense* Ching ex S. H. Wu*	Bull. Bot. Res. 9 (2): 21, f. 7. 1989
*A. lauii* Ching*	Acta Phytotax. Sinica 9(4): 360. 1964
***A. loriceum* Christ**	Index Filic. fasc. 2: 119
***A. matsumurae* Christ ex Matsumura**	Bot. Mag. Tokyo 24: 241. 1910
*A. neomultijugum* Ching ex S. H. Wu*	Bull. Bot. Res. 9 (2): 21, f. 6. 1989
*A. pseudowrightii* Ching*	Acta Phytotax. Sinica 9 (4): 360. 1964
*A. serratissimum* Ching ex S. H. Wu*	Bull. Bot. Res. 9 (2): 20. 1989
*A. subcrenatum* Ching ex S. H. Wu*	Bull. Bot. Res. 9 (2): 19, f. 5. 1989.
*A. taiwanense* Ching ex S. H. Wu*	Bull. Bot. Res. 9 (2): 18, f. 3. 1989
***A. wrightii* Eaton ex Hook.**	Sp. Fil. 3: 113, t. 182. 1860
*A. wrightioides* Christ*	Bull. Acad. Int. Géogr. Bot. 11: 238. 1902

## Material and methods

Extensive field investigation and careful examination of specimens of *Asplenium wrightii* complex from 21 herbaria (CDBI, CSH, CZH, GH, GXMG, GZTM, HGAS, HUST, HZ, IBK, IBSC, IMC, JIU, JJF, L, MO, NAS, NA, NY, PE and SYS; abbreviations follow Thiers in Index Herbariorum available at http://sweetgum.nybg.org/science/ih/) and our own collections, as well as the study of protologues and other related literature(Lin and Viane 2013; Pham 1999) were carried out.

Rhizome scales were soaked in distilled water for 24 hours and then mounted on glass slides. The morphology of rhizome scales was observed and photographed using a stereo light microscope (LEICA S8APO).

**Figure 1.**
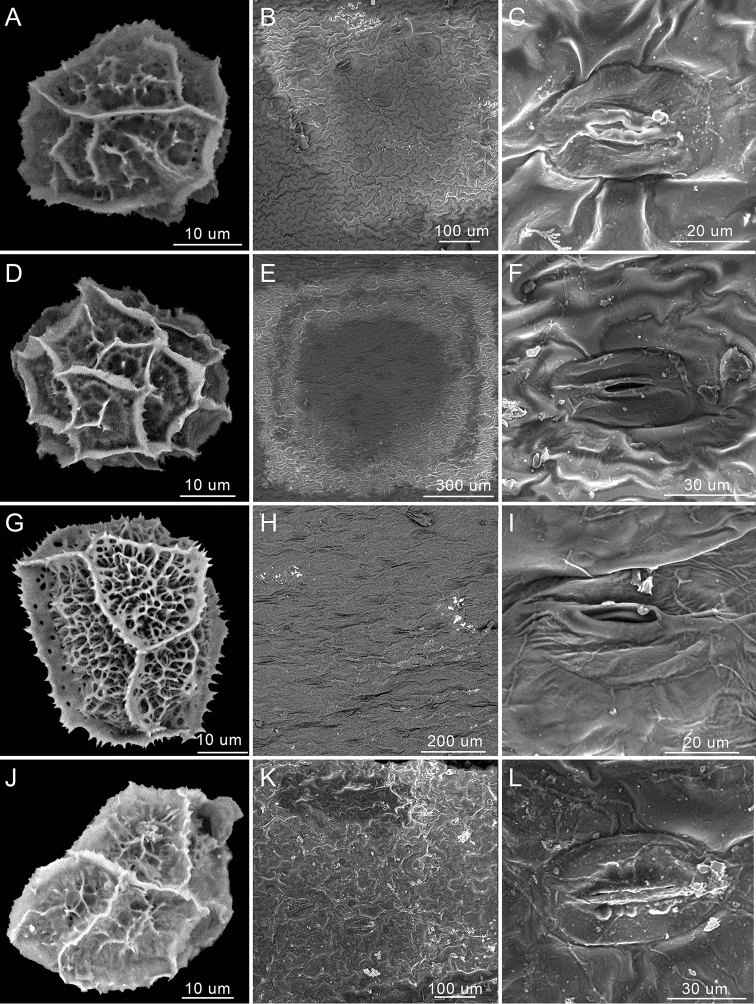
Scanning electron micrographs of spore and stomata of *A. alatulum*, *A. subcrenatum* and *A. wrightii***A–F***A. wrightii***G–I***A. subcrenatum***J–L***A. alatulum*.

**Figure 2.**
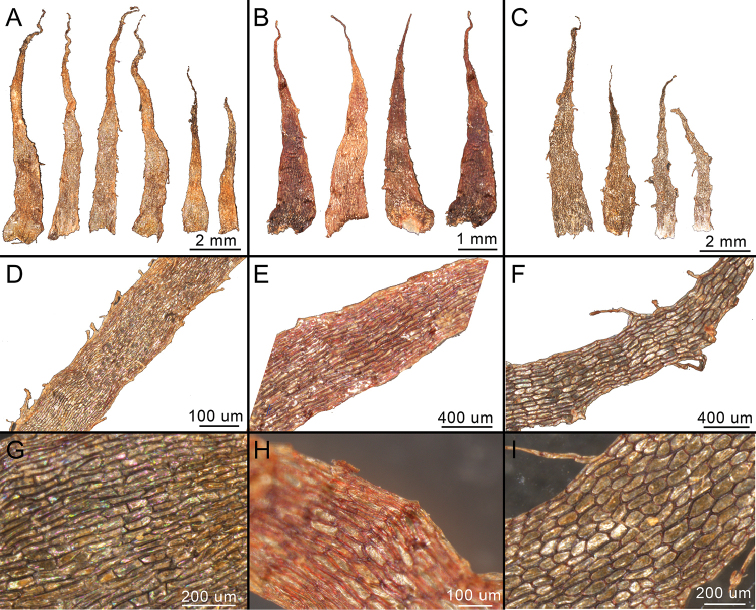
Scales of *A. alatulum*, *A. subcrenatum* and *A. wrightii*. **A, D, G***A. wrightii***B, E, H***A. subcrenatum***C, F, I***A. alatulum*.

Scanning Electron Microscope (SEM) images were taken of the spores and stomata of *Asplenium alatulum*, *A. subcrenatum* and *A. wrightii*. Spore and pinna samples, obtained from herbarium specimens, were mounted on specimen tabs and then coated with platinum in a sputter coater. Observations were conducted using a JSM-633OF SEM (JEOL Ltd., Akishima, Tokyo, Japan) scanning electron microscope with 10 kV at Sun Yat-Sen University, Guangzhou, China (Figs 1, 2).

The ImageJ software (Rasband 1997–2017) was utilised for measurement on SEM micrographs.

## Results

Though the density and shape of stomata are similar amongst *Asplenium alatulum*, *A. subcrenatum* and *A. wrightii*, the size of stomata is distinguishable amongst the three species. *Asplenium alatulum* has the largest stomata (60–70 μm in length and 40–45 μm in width, Fig. 1B, C, E, F), while the other two species *A. subcrenatum* and *A. wrightii* have similar size of stomata (45–65 μm in length and 25–35 μm in width, Fig. 1H, I, K, L). In addition, the rhizomes, scales, rachis wings, pinna pairs, pinna margins and perispores of the three species are very diverse, but relatively stable within species in morphology (Figs 1, 2, 4, 5, 6). Our results confirmed that *A. alatulum* and *A. subcrenatum*, previously treated as synonyms of *A. wrightii*, should be reinstated as distinct species. *Asplenium subcrenatum* is not restricted to their type locality, but it has an extensive geographical distribution in south-western China and northern Vietnam.

### A key to *A. alatulum*, *A. subcrenatum* and *A. wrightii*

**Table:** 

1	Stipes and rachises densely scaly, scales reddish-brown, pinna margins almost entire to crenate-sinuate, mainly occurs in limestone areas	***A. subcrenatum***
–	Stipes and rachises scaly, scales brown to dark brown, pinna margins serrate to coarsely dentate, mainly occurs in acid soil	**2**
2	Rhizomes erect to decumbent, scale cells oblong, rachises with broad lateral wings, pinnae 10–15 pairs	***A. alatulum***
–	Rhizomes erect, scale cells quadrangle, rachises only winged towards apex, pinnae (12–)17–25(–34) pairs	***A. wrightii***

### Taxonomic treatment

#### Asplenium alatulum

Taxon classificationPlantaePolypodialesAspleniaceae

Ching, Acta Phytotax. Sin. 9(4): 359. 1964.

230A5D19-1A5F-5651-8C54-2771D1A5F30F

##### Type.

**Figure 3.**
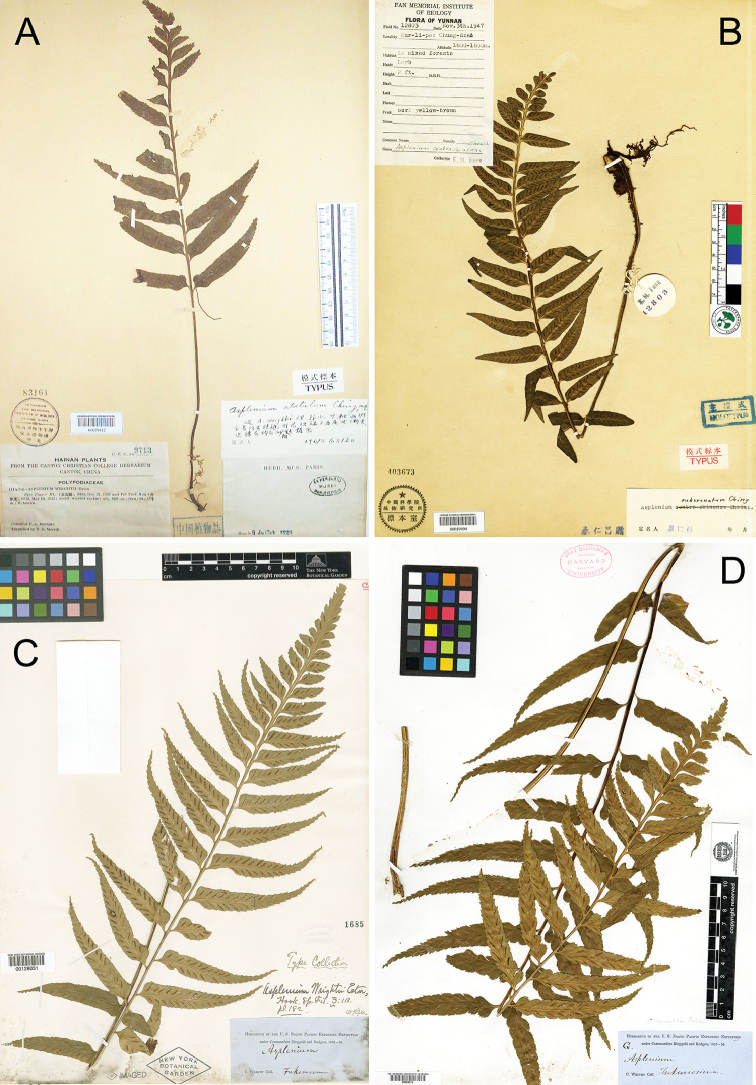
Type of *Asplenium alatulum* Ching (**A** barcode PE00059412), *A. subcrenatum* Ching ex S.H.Wu (**B** barcode PE00059494), *A. wrightii* Eaton ex Hooker (**C** barcode NY00128031) and isotype of *A. wrightii* GH (**D** barcode GH00020612).

China. **Hainan**: Five-Finger Mountain, 19 May 1922, *F.A.McClure 9713* (holotype: PE (PE00059412 [image!])). Fig. 3A.

##### Description.

**Figure 4.**
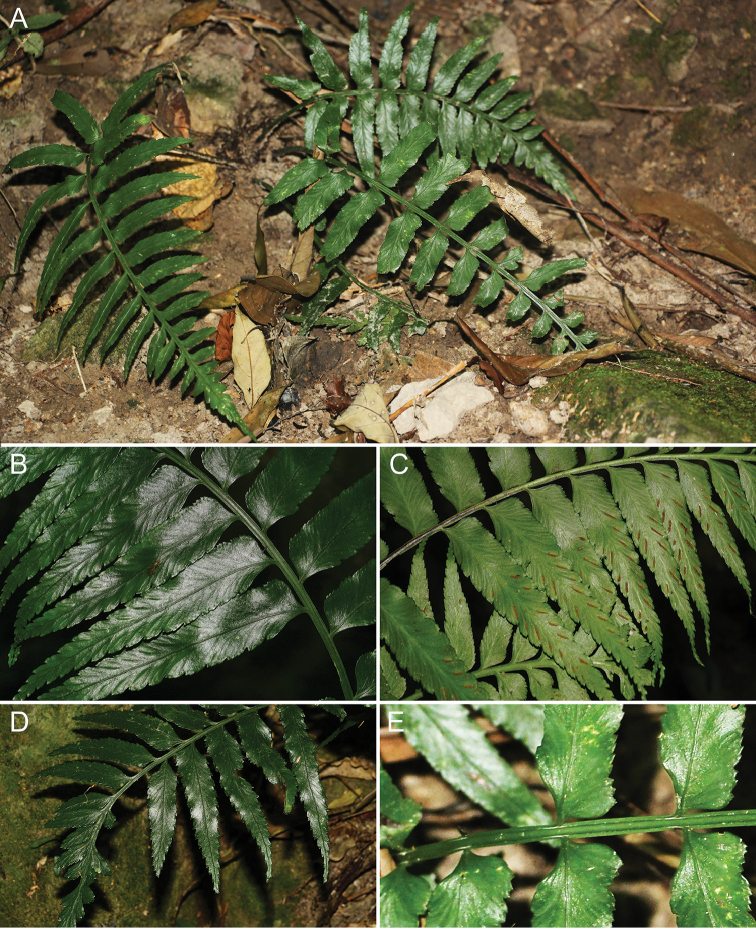
*Asplenium alatulum***A** Habitat and habit **B** Portion of lamina showing adaxial view of pinna **C** Portion of lamina showing abaxial view of pinna **D** Adaxial view of upper portion of lamina **E** Lower portion of rachis showing wide lateral wings.

Plants up to 50 cm tall. Rhizomes erect to decumbent, densely scaly; scales dark brown, narrowly lanceolate or lanceolate-ovate, 5–9 × 0.4–0.8 mm, denticulate glandular margin or long fibrillose (Fig. 2C, F, I). Fronds tufted; stipe dull to semi-shiny, greyish-green to brown or stramineous-green, 12–25 cm, sparsely scaly, scales similar to those on rhizome; lamina triangular-ovate to elliptic, (15–)18–25(–30) × (10–)12–18(–22) cm, base truncate, apex acute, 1-pinnate (Fig. 4A); pinnae 10–15 pairs, basal pinnae subopposite, others alternate, at an angle of ca. 60°–70° to rachis, with stalks (1–)2–4 mm, lower pinnae not reduced, suprabasal pinnae falcate-lanceolate, (5–)6–10(–12) × (0.8–)1.2–1.8(–2.0) cm, base asymmetrical, acroscopic side truncate at an angle of (55°–)65°–75(–85°) to costa, basiscopic side cuneate, becoming decurrent on rachis in apical part of lamina, margin serrate to dentate, apex acuminate (Fig. 4B, C, D). Veins (1 or) 2-forked, with terminal hydathode. Fronds papery, brownish-green when dry, subglabrous; rachis brown to greyish-green or stramineous-green, subglabrous, terete abaxially, with greyish-green lateral wings (Fig. 4E). Sori linear, (2–)5–9(–12) mm, on acroscopic veinlets, medial to supramedial (Fig. 4C); indusia greyish-brown to dark brown, linear, papery, margin entire, opening towards costa, persistent. Spores with average exospore length 40–45 μm, perispore cristato-alate (Fig. 1G).

##### Distribution and habitat.

*Asplenium alatulum* is apparently restricted to China. It grows on rocks in ravines of broad-leaved forests at an elevation of ca. 500–1600 m. (Fig. 4A).

##### Additional specimens examined.

**China. Hainan**: Wuzhishan, Shuiman Village, elev. 870 m, 18°54'10.6"N, 109°41'15.6"E, 7 Apr 2016, *K.W.Xu 107* (SYS!); the same locality, elev. 1550 m, 20 Dec 2010, *X.P.Wei & R.Wei WXP113* (PE-2286681!).

##### Note.

Though *Asplenium alatulum* was thought to be an endemic species to the Hainan Island before its synonymisation with *A. wrightii* by Lin and Viane (2013), the morphological distinction between *A. alatulum* and its closely-related species was obscure due to the insufficient field investigations. In the protologue, Ching and Wang (1964) emphasised its small size, the short pinnae, the crenate-serrate pinna margins and whole rachis with lateral wings and stated that *A. alatulum* is markedly different from *A. wrightii*. However, Lin and Viane (2013) recognised that the plant and pinna size and margin shape are variable in the *A. wrightii* complex and included *A. alatulum* in the synonymy of *A. wrightii*. Recently, our study, based on specimen examination and recent field observations of the two species, supports their distinction.

*Asplenium alatulum* is distinct from *A. wrightii* in having erect to decumbent rhizome (vs. erect rhizome), oblong cells of scales (Fig. 2C, F, I) (vs. quadrangle cells of scales, Fig. 2A, D, G), winged rachis (vs. winged towards apex) and fewer pinna pairs. Phylogenetically, our earlier molecular work resolved *A. alatulum* in a distinct clade, sister to the clade containing *A. wrightii* and A. × shikokianum (Xu et al. 2020). One accession from the Taiwan Island was nested within *A. alatulum*, indicating that this species should also be distributed in Taiwan.

#### Asplenium subcrenatum

Taxon classificationPlantaePolypodialesAspleniaceae

Ching ex S.H.Wu, Bull. Bot. Res. 9(2): 86, f. 5. 1989.

D809EA80-4088-51DC-BE9F-063530D7450A

##### Type.

China. **Yunnan**: Malipo, Chung-dzai, in mixed forest, elev. 1600–1800 m, 3 Nov 1947, *K.M.Feng 12803* (holotype: PE (PE00059494 [image!])). Fig. 3B.

##### Description.

**Figure 5.**
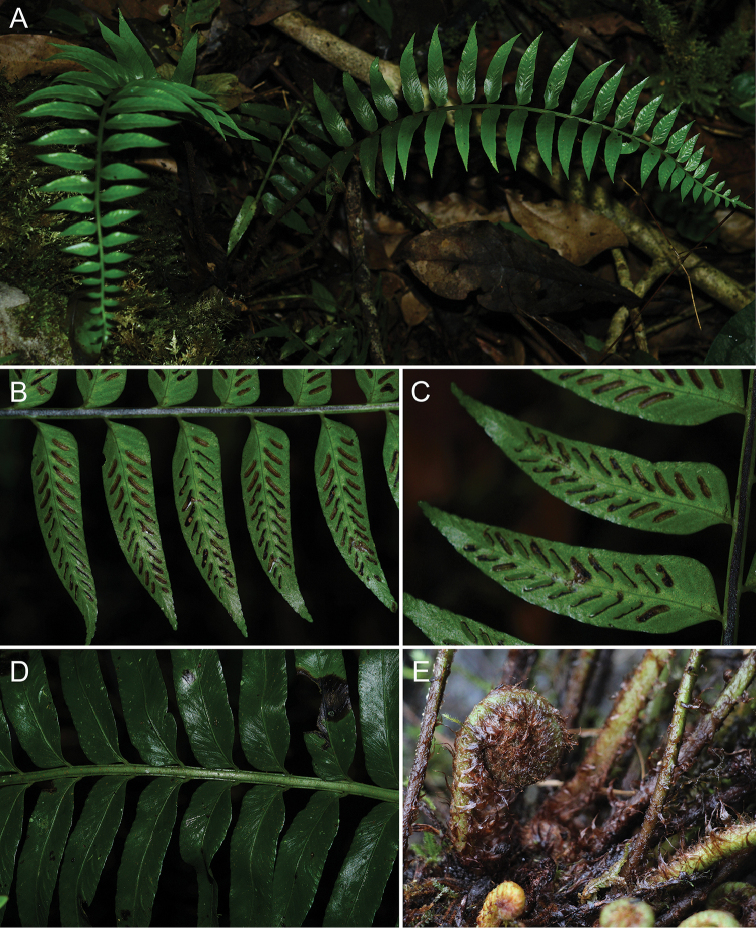
*Asplenium subcrenatum***A** Habitat and habit **B** Portion of lamina showing abaxial view of pinna **C** Abaxial view of pinna showing the serrate to coarsely dentate margin **D** Adaxial view of upper portion of lamina **E** Lower portion of stipes showing the reddish-brown scales.

Plants up to 30–55(–70) cm tall. Rhizomes erect to decumbent, densely scaly; scales reddish-brown, narrowly triangular, 4–8 × 0.7–1.1 mm, margin near entire (Figs 5E, 2B, E, H). Fronds tufted; stipe stramineous-green or reddish-brown, (10–)15–20(–25) cm, base densely scaly, scales reddish-brown, fibrillose or similar to those on rhizome; lamina oblong-lanceolate, (12–)15–40(–50) × (5–)8–15(–17) cm, base truncate, apex acute, 1-pinnate; pinnae (15–)18–25(–30) pairs, basal pinnae subopposite, others alternate, at an angle of ca. 60°–80° to rachis, with stalks (1–)2–3 mm, lower pinnae slightly reduced, suprabasal pinnae falcate-lanceolate, (3–)6–10 × (0.6–)1–1.5 cm, base asymmetrical, acroscopic side truncate at an angle of (40°–)45°–60(–70°) to costa, basiscopic side cuneate, becoming decurrent on rachis in apical part of lamina, margin almost entire to crenate-sinuate, apex acuminate (Fig. 5B, C, D). Veins (1 or)2-forked, with terminal hydathode. Fronds papery, dark green when dry, subglabrous; rachis reddish-brown to stramineous-green, densely scaly to subglabrous, scales similar to those on stipe, terete abaxially, winged towards apex. Sori linear, (2–)3–8(–10) mm, usually on acroscopic veinlets, medial (Fig. 5C); indusia greyish-brown to dark brown, linear, papery, margin entire, opening towards costa, persistent. Spores with average exospore length 40–45 μm, perispore cristato-alate.

##### Distribution and habitat.

*Asplenium subcrenatum* is distributed in China and Vietnam. It grows as an epiphyte on tree trunks or occurs on rocks by stream-sides in the evergreen broad-leaved forests of limestone areas (Fig. 5A).

##### Additional specimens examined.

**China. Gauangxi**: Jingxi County, Xinjing Village, Bahong, 23°07'05.56"N, 106°30'24.53"E, 31 Oct 2010, *L.B.Zhang, H.He & Y.Wang 5492* (MO!); Nandan County, Mangchang Village, Lala, 25°10'24.92"N, 107°23'16.26"E, 12 Oct 2010, *L.B.Zhang, H.He & Y.Wang 5492* (MO!); **Guizhou**: Libo County, Jialiang Village, 18 Oct 2018, *JSL6678* (CSH!); Libo County, Shuili Xiang, Shangshuizan, on a dry mountain with mixed pine and broad-leaved forest, 25°28'46"N, 107°47'47"E, 8 Jun 2016, *L.B.Zhang et al.9193* (MO!); Libo County, Wong’ang, Dongduo, elev. 780 m, 16 Sep 2007, *L.B.Zhang 472* (MO!); Guiding County, Houchangbao Xiang, on cliffs by a stream, elev. 1100 m, 26°14'47"N, 107°12'37"E, 10 Jun 2016, *L.B.Zhang, Y.F.Duan, N.T.Lu & X.Y.Miao 9250* (MO!); **Yunnan**: Xichou County, Fadou Village, Xinjing, elev. 1800 m, 10 Jun 2013, *Y.H.Yan YN250* (CSH-0046594!); the same locality, 9 Jan 1962, *S.G.Wu4222-62* (PE-00912376!); Malipo County, elev. 1100 m, 21 Jan 1940, *C.W.Wang 86341* (PE-00912378!); Xiajinchang Village, Huangjinyin, elev. 1416 m, 22°07'28.89"N, 104°51'11.15"E, 29 Oct 2015, Fan 13883 (SYS!); the same locality, 29 Oct 2015, Fan 13884 (SYS!); the same locality, 11 Dec 2015, the same locality, *Xu TTJ-YN-031* (SYS!); 11 Dec 2015, *Xu TTJ-YN-032* (SYS!); Tianbao County, Tianbao Village, Bajiaoping, elev. 1135 m, 22°58.6607'N, 104°50.8035'E, 30 Oct 2015, Fan 13884 (SYS!); Maguan County, Bazhai, Lvditang, 7 Apr 2017, *X.C.Zhang et al. 8219* (PE-02236348).

**Vietnam. Ha Giang**: Yen Minh District, Lao Va Chai Municipality, vicinities of Chi Sang Village, elev. 1450 m, 23°06'20"N, 105°04'25"E, 09 Dec 2005, *Averyanov, Leonid V. HAL8420* (MO-3136047!); Vi Xuyen District, Tung Ba Community, Khau Ca Nature Reserve, Hom Mountain, elev. 900 m, 13 Dec 2013, *L.B.Zhang, L.Zhang & L.T.Ngan 6966* (MO!); Quan Ba District, Nghia Thuan Community, Bat Dai Son Nature Reserve, 10 Dec 2013, *L.B.Zhang, L.Zhang & L.T.Ngan 6882* (MO!).

##### Note.

Just like *Asplenium alatulum*, a comprehensive taxonomic study of *A. subcrenatum* was scarce after this species was described. Lin and Viane (2013) treated it as a synonym of *A. wrightii*, based only on macromorphological data. Both our molecular data (Xu et al. 2020) and micromorphological data in this study (Figs 1, 2) support the distinction of *A. subcrenatum*.

*Asplenium subcrenatum* is different from *A. wrightii* in having its stipe and rachis covered with reddish-brown scales (Fig. 2B, E, H) (vs. brown to dark brown scales, Fig. 2A, D, G), scale margins nearly entire (vs. glandular margins or long-fibrillose) and pinna margins almost entire to crenate-sinuate (vs. serrate to coarsely dentate). Meanwhile, the perispores of *A. subcrenatum* are different from those of *A. wrightii*. The folds of *A. subcrenatum* are cristato-alate and areolofenestrate and the margins of the folds are echinulate, while the folds of *A. wrightii* are cristato-alate and imperforate and the margins of the folds are echinulate or approximately laevigate.

Geographically, *Asplenium subcrenatum* was only known from the type locality Malipo County and Yanshan County, southern Yunnan, China (Wu 1999). Our study, based on specimen investigations and the field survey, shows that this species is mainly distributed in limestone areas of south-western China and northern Vietnam, while *A. wrightii* is widely distributed in acidic soils in southern China and Japan and it might occur in Korea.

#### Asplenium wrightii

Taxon classificationPlantaePolypodialesAspleniaceae

Eaton ex Hooker, Sp. Fil. 3: 113. pl. 182. 1860.

2F8AFE4E-FBFB-5A10-87EF-EB580AFE2515

Asplenium duplicatoserratum Ching ex S.H. Wu, Bull. Bot. Res., Harbin 9(2): 86, f. 4. 1989.
Asplenium fujianense Ching ex S.H. Wu, Bull. Bot. Res., Harbin 9(2): 21, 88–89, f. 7. 1989.
Asplenium laui Ching, Acta Phytotax. Sin. 9(4): 360–361. 1964.
Asplenium neomultijugum Ching ex S. H. Wu, Bull. Bot. Res. 9 (2): 21, f. 6. 1989.
Asplenium pseudowrightii Ching, Acta Phytotax. Sin. 9(4): 360. 1964.
Asplenium serratissimum Ching, Bull. Bot. Res., Harbin 9(2): 87. 1989.
Asplenium taiwanense Ching ex S.H. Wu, Bull. Bot. Res., Harbin 9(2): 85–86, f. 3. 1989.
Asplenium wrightioides Christ, Bull. Acad. Int. Géogr. Bot. 11(153–154): 238–239. 1902.

##### Type.

Japan. **Kagoshima**: Ryukyu Islands, 1853–1856, *C. Wright, #s.n.* (holotype: NY (NY-00128031 [image!], isotype: GH (GH00020612 [image!])). Fig. 3C, D.

##### Description.

**Figure 6.**
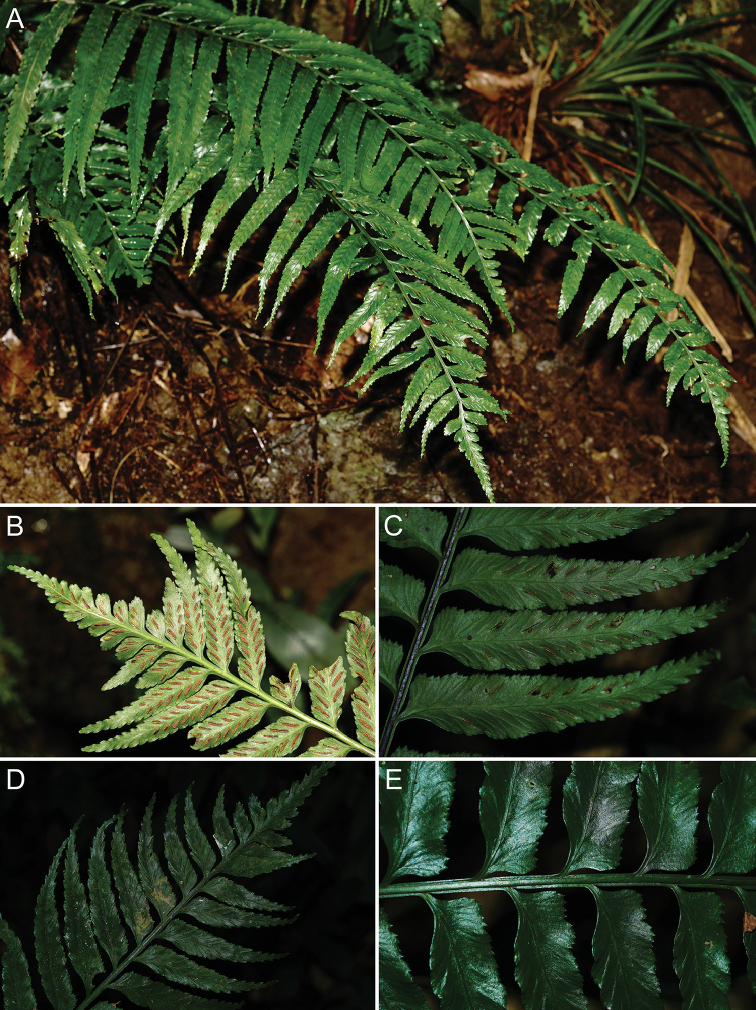
*Asplenium wrightii***A** Habitat and habit **B** Abaxial view of upper portion of lamina **C** Abaxial view of pinna showing the almost entire to crenate-sinuate margin **D** Adaxial view of upper portion of lamina **E** Upper portion of rachis showing the lateral wings.

Plants 35–70(–100) cm tall. Rhizome erect, short, scaly; scales brown to dark brown, lanceolate-triangular, 5–12 × 0.6–1.2 mm, denticulate glandular margin or long fibrillose. Fronds tufted; stipe greyish-green to brown, (18–)23–29(–31) cm, base densely scaly, scales brown, lanceolate to linear-lanceolate, (4.6–)6.1–7.8(–8.8) mm long, with multicellular hairs, subglabrous towards apex; lamina ovate-lanceolate to lanceolate, (19–)39–70(–88) × (9–)17–27(–35) cm, base truncate, apex acute, 1-pinnate; pinnae (12–)17–25(–34) pairs, basal pinnae subopposite, others alternate, at an angle of ca. 50°–60° to rachis, with stalks (2–)4–8 mm, lower pinnae slightly reduced, suprabasal pinnae narrowly oval-lanceolate and often falcate, (6–)9.1–13(–20) × (0.9–)1.2–1.8(–2.5) cm, base asymmetrical, acroscopic side truncate at an angle of (40°–)55°–75°(–85°) to costa and often auriculate, basiscopic side cuneate, at an angle of (20°–)30°–40°(–60°), becoming decurrent on rachis in central part of lamina, margin serrate to coarsely dentate, apex acuminate. Veins (1 or)2-forked, with terminal hydathode (Fig. 6B, C, D). Fronds papery, green to brownish-green when dry, subglabrous; rachis dull green to reddish-brown, terete abaxially, winged towards apex. Sori linear, (3–)6–10(–12) mm, on acroscopic veinlets, medial (Fig. 6B, C); indusia brown, linear, papery, opening towards costa, persistent. Spores with average exospore length 32–45 μm, perispore cristato-alate.

##### Distribution and habitat.

*Asplenium wrightii* is commonly distributed in China and Japan and it might occur in Korea. This species is found in damp valleys under evergreen broad-leaved forests where it grows as a lithophyte by streams (Fig. 6A).

##### Additional specimens examined.

**China. Anhui**: Qimen County, Xifengsi, Nov. 1957, *M.B.Deng 5308* (HZ-028581); Jing County, Dingxi Village, Suhong, 22 Sep 1959, *0431* (NA-S00153661); Tongling County, Xiaokeng, Jun 1985, *X.L.Liu 85053* (HUST-00008926); **Chongqing**: Pengshui County, Hanjia Village, elev. 370 m, 29°25'137.98"N, 108°18'19.1"E, 16 Oct 2012, *Pengshui expedition 500243-003-167* (IMC-0003680); Yangshan County, Nanling Nature Reserve, Xinerkeng, 29 Sep 2007, *X.L.Zhou & H.F.Chen 785* (HUST-00008767); **Fujian**: 22 Jul 2015, *ZXL05538* (CSH-0101629); 10 Apr 2014, *H.Shang & Y.F.Gu SG134* (CSH-0034578); Wuyishan, elev. 750 m, 15 Aug 2011, *X.F.Zeng 11215* (CZH-0006554); Zhaoan County, Wushan, 12 Apr 2015, *X.F.Zeng ZXF20041* (CZH-0012412); Nanping City, Mangdangshan, 17 Feb 1999, *G.S.He 9643* (PE-00913318); Jianning County, 20 Nov 1977, *Z.Y.Li 10619* (PE-0913321); Jiangle County, Longxishan, 27 May 1991, *Longxishan expedition 0359* (PE-01555190); **Guangxi**: Lingyun County, Yuhong Village, Donglan, elev. 1303 m, 24°24'13.19"N, 106°29'03.17"E, 16 Aug 2013, *Lingyun expedition 451027130816103* (GXMG-0117634); Longshen County, Heping Village, elev. 615 m, 25°41'12.10"N, 110°03'22.40"E, 6 Mar 2013, *Longshen expedition 450328130306038LY* (IBK-00362003); Xingan County, Maoershan, 5 Oct 2007, *L.Wu & X.X.Xu 1042* (HUST-00019436); Sanjiangdongzu County, Sanxingpo, Dudong Village, elev. 700 m, 18 Dec 2007, *X.X.Xu & L.C.Qin 255* (HUST-00011550); **Guangdong**: Heping County, Daba Village, Dafukeng, 2 Jan 2007, *C.M.Tan et al. Y06630* (HUST-00004651); Lechang County, 23 Nov 1931, *Z.Huang 31490* (IBK-00035278); the same locality, 21 Jun 1942, *S.Q.Chen1611* (IBK-00035299); Boluo County, Luofushan, 18 Aug 1930, *N.Q.Chen 41631* (IBK-00035279); Xinyi, 13 Aug 1931, *Z.Huang 31164* (IBK-00035285); Dawuling, 4 Aug 2003, Y.H.Yue et al. 1568 (PE-01784537); 21 Mar 1931, *Z.Huang 31756* (PE-00913341); Ruyuan, Daxiagu, 2 Aug 2005, *B.R.Liu 05100* (PE-01785865); Yingde, Shimentai Nature Reserve, Oct 2001, *Y.H.Yue & F.W.Xing 13265* (PE-01784538); Mei County, Jiaying, 4 Aug 1932, Tsang,*W.T. 21466* (PE-00913338); **Guizhou**: Yongshun County, Xiaoxi Village, elev. 621 m, 28°82'21.10"N, 110°25'11.00"E, 14 Jan 2014, *D.G.Zhang zdg9949* (JIU-04159); Liping County, Pingjia, elev. 670 m, 16 Dec 2007, *X.X.Xu & L.C.Qin 349* (HUST-00011558); Libo County, Maolan Nature Reserve, 25 Apr 2015; *X.C.Zhang 7259* (PE-02051530); Shiqian County, Qiangyang Village, Longdong, 30 Jul 1988, *Wulingshan expedition 2355* (PE-01557785); Jiangkou County, 30 Aug 1986, *B.Bartholomew et al. 595* (PE-00913309); Jianhe County, Nanjiaqu, 30 Apr 1992, *F.Wang 651* (HGAS-055247); Jinping County, Gaodengpo, 27 Nov 1991, *fern expedition 91474* (HGAS-055248); Rongjiang County, Shuiwei Shuizu Village, 15 Oct 2014, *Wei et al. WYG036* (CSH-0043860); Shibing County, Maohao Village, 30 May 2016, *D.Y.Zhou 522623160530476LY* (GZTM-0066044); Danzhai County, 18 Oct 2012, *Hou GZDZ201210180003* (GYBG-0009834); **Hunan**: Sangzhi County, Bamaoxi Village, 21 Oct 2014, *X.L.Zhou et al. ZXL09673* (CSH-0045103); Baojing County, Baiyunshan Nature Reserve, elev. 397 m, 28°37'51.90"N, 109°17'11.34"E, 11 Aug 2012, *X.J.Su. & H.B.Liu 433125D00030810086* (JIU-06613); Yongshun County, Xiaoxi, Daping, 12 Sep. 2009, *L.Xu 090912005* (JIU-02238); Dongzu County, Pingyang Village, Yangdongtan, elev. 300 m, 10–15 Jan 2008, *L.C.Qing & H.B.Ouyang 1080* (HUST-00012147); Shangyan, Sanxingpo, elev. 750 m, 14 Dec 2004, *L.C.Qing. & X.X.Xu 691* (HUST-00011548); Suining County, Huangsang Nature Reserve, elev. 450 m, 10 Jan 2008, *J.M.Xi & Y.B.Qin 07919* (HUST-00012150); Yanling County, Taoyuandong Nature Reserve, Jul 2008, *X.L.Zhou &Z.L.Zhu 2154* (PE-01964123); Rucheng County, Donggangling, Shuinishan, elev. 110 m, 31 Jul 2007, *L.Wu & H.B.Ouyang 88* (HUST-00008785); Pingjiang County, Sicun Village, Wudeng, elev. 500 m, 15 Dec 2007, *L.Wu & S.X.Qi W467* (HUST-00011555); Huaihua County, Bamianshan, 15 Dec 2007, *X.L.Zhou & Y.Q.Yao 1744* (HUST-00011562); **Hubei**: Laifeng County, Dahe Village, Lianghekou, elev. 590 m, 25 Jul 2013, *W.Z.Zhu BLF077* (CCAU-0002976); **Jiangxi**: Xingzi County, Wenquan Village, Xiufeng, 26 Jul 2013, *A.M.Dong 2082* (JJF00010847); Wanzai County, Jiulong Forest Park, elev. 190 m, 28°21'38"N, 114°30'47"E, 15 Aug 2013, *H.G.Ye & F.Y.Zeng LXP10-2161* (IBSC-0773610); Wuning County, Yuangkou Village, Dongkeng, elev. 400 m, 16 Oct 2012, *J.H.Zhang 2045* (JJF-00010846); Chongyi County, Fengzhou Village, Changlongao, elev. 1000 m, 8 Jul 2007, *L.Y.Wang W.181* (HUST-00008778); Jianggangshan, elev. 800 m, 10 Apr 2004, *Y.H.Yan & J.S.Zhou 3415* (HUST-00001166); **Jiangsu**: Yixing, Longchi, Shuizhushan, 1956, *Liu & Huang 2874* (NAS-00091223); **Sichuan**: Emeishan, 15 Sep 1963, *G.X.Xing & K.Y.Lang 1788* (PE-13307); the same locality, 21 Sep 1979, *Z.R.Wang C122* (PE-96543); Zhulian County, 1 Jun 1978, *X.R.Kong 5204* (CDBI-003584); **Taiwan**: Wulai, Fushan-Hapen, 13 Oct 1984, *Y.Tateishi et al. 20492* (PE-01451621); Takao, 27 Jan 1939, *M.Tagawa 2049* (PE-00913326); *W.Hancock 141* (PE-00913329); Taito, 4 Mar 1940, *M.Tagawa 3168* (PE-00913330); Huanlian County, Changliang, 8 Jun 2001, *T.C.Chen 11303* (PE-01451613); Taibei County, Lujiaokengxi, 28 Jan 2000, *T.C.Chen 10393* (PE-01451614); **Zhejiang**: Tonglu County, Luci, Changzhou, 8 Nov 1989, *L.Hong s.n.* (HZ-028576); Shouchang Zhen, 19 Jun 1959, *Zhejiang expedition 27803* (HZ028578); Taishun County, Liguang, Huangshikeng, 27 Nov 1958, *Anonymous 23818* (HZ-028579); Lishui, Suichang Nature Reserve, elev. 780 m, 25°59'11"N, 116°25'11"E, 5 Jun 2012, *Q. Tian TQ01773* (CSH-0002430); 25 Nov 2015, *H.J.Wei JSL3466* (CSH-0110219); 29 Jul 1958, *Shan 5578* (PE-00913289);

**Japan. Kyushu**: Chikushi-yabakei, Nakagawa-cho, Chikushi-gun, Fukuoka Pref., 27 Mar 1975, *T.Nakaike et al. 105* (PE-01708808); Is. Yakushima (Kagoshima Pref.), from Kurio to Segiri through Ookonotaki, elev. 30–100 m, 16 Jul 1979, *T.Yamazaki et al. 2327* (PE-01708809); Kagoshima Pref., Okuchi-shi, Jusso, 6 Sep 1959, *Tetsuji Yamanaka s.n.* (PE-01964369); Prefecture Kagoshina, Yakushina Island, Yaku-cho, Tainoko-gawa River, 10-11 Oct 1977, *Boufford, David Edward 20118* (MO-3136075); Along trail in gorge, foot of Mt. Awa. Motobu Peninsula, Okinawa Island, 10 Dec 1953, *Walker, EH 7614* (L-3508507); **Honshu**: Owase City, Kuki, Namera Valley, 12 Nov 1991, *K.H.Shing 31* (PE-01708810); Pref. Mie,Kuki, Omse-shi, 5 Oct 1971, *Kato, Masahiro-259* (MO-3136077); Pref. Schizouka: Aono, Minamiizu-cho, Kamo-gun, 30 Dec 1972, *T.Nakaike 5* (L-3508506); **Shikoku**: Nakaohsaka Ohsakadani, Nakatosa Town, Takaoka Distr. Kouchi Pref., elev. 60–80 m, 33°18'48"N, 133°23'48"E, 6 Jan 2012, *Taku Miyazaki 1201164* (PE-02002704); Yatabe, Nakatosa Town, Takaoka District, Kouchi Pref., elev. 40–60 m, 33°15'70"N, 133°15'37"E, 3 Jan 2007, *T.Miyazaki 0701137* (PE-01963957); Takanoyama-rindou Yamauchi, Nakatosa Town, Takaoka Distr. Kochi Pref., elev. 60–90 m, 33°17'04"N, 133°12'18"E, 27 Mar 2009, *Taku Miyazaki 0903138* (PE-01963829); Ohkawauchi Kaminokae, Nakatosa Town, Takaoka District, Kouchi Pref., elev. 20 m, 33°16'67"N, 133°14'15"E, 29 Dec 2006, *Taku Miyazaki 0701025* (PE-01962310).

##### Note.

*Asplenium wrightii* is described based on the type material from Japan and it is widely distributed in China. The morphology of this species is variable, which resulted in the taxonomic chaos amongst *A. wrightii* and its closely-related species. Several ploidy levels (e.g. tetraploid, octoploid, decaploid and dodecaploid) have been reported for the *A. wrightii* complex, but no correlation was found between ploidy levels and morphological characters within the complex (Mitui 1967; Wang 1988; Lin and Viane 2013). Asplenium × shikokianum is a natural hexaploid hybrid between octoploid *A. wrightii* and tetraploid *A. ritoense* and Asplenium × wangii is another hybrid between *A. wrightii* and *A. bullatum*. These hybrids are usually uncommon in terms of their distributions where their parents grow together (Kuo 1988; Lin and Viane 2013). Due to hybridisation and polyploidisation, there are a large number of recognised species complexes with ambiguous boundaries between species in Aspleniaceae (Reichstein 1981; Dyer et al. 2012). More comprehensive taxonomic studies are still needed to elucidate the species delimitation in this species complex.

*Asplenium wrightii* was also documented in Vietnam (Pham 1999; Lin and Viane 2013). However, we checked specimens identified as *A. wrightii* from Vietnam and found none of these specimens is true *A. wrightii*. It is possible that the name *A. wrightii* has been used erroneously for species *A. subcrenatum* in Vietnam.

## Supplementary Material

XML Treatment for Asplenium alatulum

XML Treatment for Asplenium subcrenatum

XML Treatment for Asplenium wrightii
